# Piezoelectric stepped-plate resonators vibrating at lateral modes for direct viscosity determination in liquids

**DOI:** 10.1038/s41378-025-01135-7

**Published:** 2026-04-07

**Authors:** Linya Huang, Dejiang Lu, Xiangguang Han, Heping Wu, Wei Li, Gang Niu, Kaifei Wang, Ping Yang, Wei Ren, Libo Zhao

**Affiliations:** 1 Electronic Materials Research Laboratory, Key Laboratory of the Ministry of Education, School of Electronic Science and Engineering, Xi’an Jiaotong University, Xi’an, China; 2 State Key Laboratory for Manufacturing Systems Engineering, International Joint Laboratory for Micro/Nano Manufacturing and Measurement Technologies, Xi’an Jiaotong University, Xi’an, China; 3 School of Instrument Science and Technology, Xi’an Jiaotong University, Xi’an, China; 4 State Industry-Education Integration Center for Medical Innovations, Shaanxi Innovation Center for Special Sensing and Testing Technology in Extreme Environments, Shaanxi Provincial University Engineering Research Center for Micro/Nano Acoustic Devices and Intelligent Systems, Xi’an Jiaotong University, Xi’an, China; 5 Department of Emergency, The First Affiliated Hospital of Xi’an Jiaotong University, Xi’an, China

**Keywords:** Electrical and electronic engineering, Nanoscience and technology

## Abstract

Precise viscosity measurement in liquid media remains a critical challenge for micromachined resonant sensors. This primarily results from the inherent coupling between viscosity and density in hydrodynamic interactions, which limits independent quantification of viscosity. This work presents an aluminum nitride (AlN) piezoelectric microresonator vibrating at in-plane lateral mode for direct viscosity sensing in liquids. The resonator features cantilevered dual-plate structure with a wide step to leverage width-dependent effects on resonant frequency and quality factor. Through fluid-structure interaction modeling, the resonator is optimized to enhance vibrational characteristics while increasing linearity with respect to liquid viscosity. In experiments, the resonator incorporates fully electrical interfaces by combining self-actuation and self-sensing capabilities under liquid immersion. Furthermore, a significantly linear relationship between the quality factor and liquid viscosity is demonstrated. This linearity enables direct viscosity quantification through simultaneously measuring the resonant frequency and quality factor, which eliminates the need for prior density calibration required by conventional methods. The fabricated resonator achieves a mean absolute relative deviation of 2.65% with a maximum stability deviation of 3.43%. These results establish microplate-based laterally vibrating resonators as promising solutions for high-precision viscosity determination in compact liquid monitoring systems.

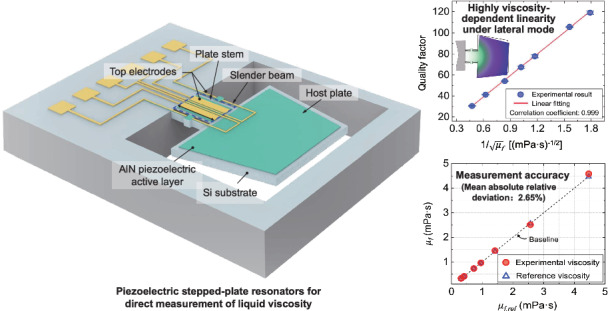

## Introduction

Viscosity quantification of liquids serves as a fundamental physical parameter in diverse industrial and biomedical domains, including chemical process monitoring, pharmaceutical formulation, and point-of-care diagnostics^1–4^. Conventional viscometry platforms, predominantly rotational rheometers and capillary viscometers, are often expensive, time-consuming and difficult in miniaturization configurations. Resonant microelectromechanical systems (MEMS) sensors offer an appealing alternative due to their online measurement capability, compatibility with integrated circuits, and potential for low-cost, high-volume fabrication^5–8^. For micromachined resonators operating in liquid environments, a vibration mode with reduced energy losses is sought. The viscous damping and hydrodynamic drag induced by the liquid impose challenges on the resonator to maintain a high quality factor and stable vibration for accurate measurement, particularly when compared to operation in gaseous environments^9^. The use of lateral vibrational modes which are also called in-plane flexural modes, offers a promising approach to enhance the quality factor of resonant sensors operating under liquid immersion^10,11^. It was previously proposed that lateral modes of the resonator are considered to yield lower viscous damping compared to out-of-plane modes, which can be attributed to shear flow rather than squeeze flow induced in the surrounding liquid. Cox et al.^12^ reported that laterally vibrating microcantilevers in aqueous glycerol achieve quality factors up to 4 times higher than those operating in fundamental out-of-plane flexural modes, which contributes to a substantial increase in mass sensitivity. Beardslee et al.^13^ presented a thermally excited cantilever operating in lateral modes for improving detection limits in liquid sensing, which exhibited a quality factor of 67 in water with reduced added fluid mass effects compared to out-of-plane flexural modes.

Although laterally vibrating resonators experience reduced fluid damping and enhanced quality factors, building a precise quantitative model for viscosity remains challenging. This challenge specifically involves modeling the relationship between viscosity and resonance parameters, particularly the quality factor and resonant frequency. This difficulty stems from the second Stokes problem^14^ that the resonator response depends on the product of liquid density and viscosity^15,16^, which constrains independent viscosity determination and often necessitates empirical fitting approaches. For instance, Riesch et al.^17^ proposed a laterally vibrating resonator under Lorentz force excitation, which served as a viscosity sensor by establishing a fitted curve between the damping factor and liquid viscosity. While numerous studies have focused on increasing the quality factor of in-plane-mode resonators in viscous liquids^18–20^, relatively few have addressed quantifying fluid properties. For specific configurations, the hydrodynamic model is available for the resonator to extract fluid density and viscosity values^21,22^. This approach is time-intensive and requires extensive experimental data post-processing. Generally, viscosity cannot be measured independently but is coupled with density calibration, which introduces larger errors than direct measurement methods. Consequently, fully leveraging the capability of enhancing the quality factor of laterally vibrating resonators for precise liquid viscosity quantification requires rigorous design and modeling.

The lateral-mode resonator has conventionally employed thermal actuation via boron-diffused heating resistors. Beardslee et al.^23^ demonstrated this approach using a hammerhead-shaped resonator with thermal excitation and piezoresistive detection, which estimated limits of detection in the sub-ppm range for gas-phase sensing. Xu et al.^24^ reported an electrothermally actuated microcantilever featuring a triangular tip for lateral vibration in liquid-phase analysis. Compared with the thermal excitation, the piezoelectric resonator employs the high electromechanical coupling of piezoelectric thin films, enabling self-actuation and self-sensing through intrinsic piezoelectric stress gradients. This mechanism significantly reduces thermal stress effects on surrounding fluids, which is beneficial for improving precision for liquid-phase measurements. However, piezoelectrically lateral-mode resonators have been limited in liquid sensing, which requires highly tailored structural designs and electrode layouts. For example, mode coupling induced by multi-mode excitation^25^ degrades modal purity and operational stability. Furthermore, the operation of resonators in lateral modes is typically characterized by higher modal stiffness and resonant frequencies than that of fundamental out-of-plane flexural modes. This condition limits the achievable mechanical strain, thereby compromising the piezoelectric self-sensing capability^18,25^. These constraints necessitate resonator designs that simultaneously optimize lateral-mode excitation efficiency and ensure electrical interfacing for compact liquid monitoring.

Here we present a piezoelectric aluminum nitride (AlN)-actuated resonator designed for lateral-mode vibration to serve as a liquid viscosity sensor. The vibrational characteristics of the resonator were analyzed through fluid-structure interaction modeling, which provided a design guideline to enhance the response linearity with liquid viscosity. By varying the dimensions of the cantilevered free end, the resonant frequency and the linear dependence of the quality factor on liquid viscosity were simultaneously optimized. Furthermore, the fabricated resonator was experimentally characterized, operating at a moderate frequency to allow self-actuation and self-sensing under liquid immersion. A highly linear relationship between the quality factor and liquid viscosity was revealed. This condition significantly simplifies the sensing configuration by establishing explicit and individual equations for viscosity measurement that are density-independent. Based on these investigations, the experiments demonstrated the high performance of the resonator in direct viscosity sensing. Such results confirm design strategies for implementing precise, miniaturized piezoelectric MEMS sensors in liquid-phase applications.

## Materials and methods

### Resonator design and modeling

**Fig. 1:**
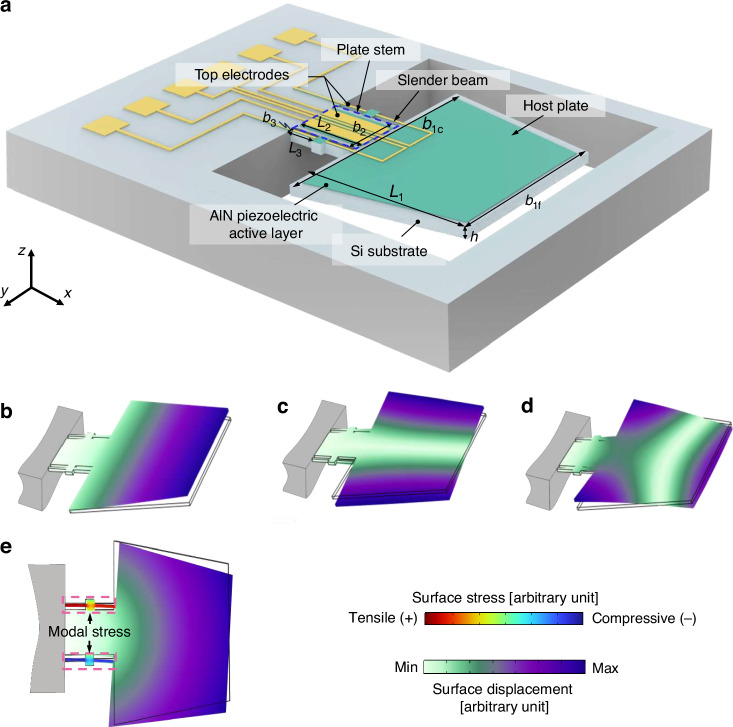
Overall design of the piezoelectric resonator featuring microcantilevered dual-plate structure with a wide step. **a** Detailed components of the resonator. **b** Mode shape of the fundamental flexural resonance under simulated vibrational displacements**. c** Mode shape of the fundamental torsional resonance under simulated vibrational displacements. **d** Mode shape of the 2nd flexural resonance under simulated vibrational displacements. **e** Mode shape of the lateral resonance under simulated vibrational displacements, also showing the modal stress on the slender beam

The microcantilevered dual-plate resonator comprises a trapezoidal host plate, a supporting plate stem, and slender beams for piezoelectric readout, as shown in Fig. 1a. The plate stem connected to the clamped side exhibits a largely reduced width compared to the host plate, which creates a wide step that enables targeted stiffness modulation. This configuration promotes in-plane compliance by facilitating *x*-*y* deformation of the trapezoidal plate while ensuring frequency separation from out-of-plane motions. The trapezoidal host plate serves as the primary fluid-interacting surface for viscosity sensing. Its narrowing free end reduces inertial mass and elevates the in-plane resonant frequency relative to conventional rectangular designs. The asymmetric mass distribution of the trapezoidal plate along the *x*-direction promotes the excitation of in-plane lateral modes by the piezoelectric drive force on the plate stem, which generates a shear-dominated flow field in the surrounding liquid. The slender beams provide piezoelectric transduction for signal readout, and partitioned blocks on both sides are designed to enhance distributed axial stress and electrical amplitude. The top electrodes are divided into sections positioned on both the plate stem and the slender beams. The actuation electrodes are located on the plate stem and are supplied with out-of-phase alternating voltages, which create a bending moment that excites the resonator into the lateral vibrational mode. The sensing electrodes on the slender beams detect the piezoelectric charge generated by the vibrational stress. Considering that the stress changes oppositely in the slender beams of the resonator under lateral mode, the sensing electrodes on each slender beam are divided into four parts. This configuration allows for an additive readout on the same side of the plate stem and a differential readout on the opposite side, thereby enhancing the electrical output amplitude of the resonator. This separated configuration of actuation and sensing electrodes is designed to minimize parasitic effects and charge crosstalk within the AlN layer during vibration.

The finite element method (FEM) simulation model was developed using COMSOL Multiphysics® v. 6.2. The model combines domains of Solid Mechanics, Pressure Acoustics, and Thermoviscous Acoustics to capture fluid-structure interaction, along with an Electrostatics module for piezoelectric effect simulation. The silicon-based resonator was modeled by the Solid Mechanics module with an elastic modulus of 170 GPa, density of 2329 kg/m^3^, and Poisson’s ratio of 0.28^26^. The Electrostatics module was coupled with the Solid Mechanics module via the piezoelectric coefficient matrix. The Pressure Acoustics module was boundary-coupled with the Thermoviscous Acoustics module to model the fluid domain. And the outer boundaries of the fluid domain were set to spherical wave radiation conditions to emulate an unbounded fluid environment. The vibrational mode shape of the stepped dual-plate microcantilever is presented in Fig. 1b–e, including the fundamental flexural mode, torsional mode, 2nd flexural mode, and lateral mode. The flexural and torsional resonances constitute out-of-plane vibrational modes, while the lateral is an in-plane mode. It is noted that the torsional and 2nd flexural modes exhibit distinctly different mode shapes compared to conventional one-dimensional cantilevers, which is a consequence of the wide-step structure of the resonator. This resonator design enables functional separation: the plate stem primarily governs vibrational stiffness while the trapezoidal plate acts on hydrodynamic interactions through mode shape displacement.

**Fig. 2:**
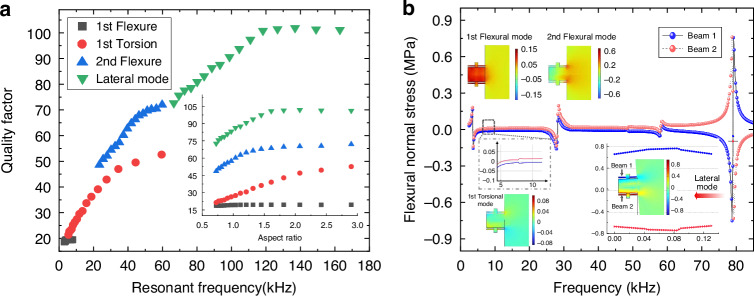
Simulation results of the microcantilever-based resonator under various out-of-plane modes and in-plane lateral modes. **a** Dependence of the quality factor on resonant frequency and aspect ratio for the resonator. **b** Flexural normal stress distribution along the slender beam for different vibrational modes of the resonator, derived from frequency-domain simulations

The initial dimensions of the stepped dual-plate microcantilever are designed with *L*_1_ = 1100 μm, *b*_1c_ = 1900 μm, *b*_1f_ = 1500 μm, *L*_2_×*b*_2_ = 350 μm × 280 μm, *L*_3_×*b*_3_ = 145 μm × 20 μm, with a uniform thickness *h* = 30 μm. Figure 2a shows the variation of quality factor with resonant frequency in cyclohexane (dynamic viscosity of 0.959 mPa·s, density of 0.779 g/cm^3^). This analysis focuses on rectangular host plate where *b*_1c_ equals *b*_1f_, with widths ranging from 500 – 2000 μm. The resonant frequency was modulated by varying the width of the rectangular plate while maintaining constant cantilever length to isolate width-dependent effects on vibrational behavior. The resonant frequency decreases with increasing plate width due to the reduced vibrational stiffness. The quality factor versus aspect ratio (dominant scale of width/length) is displayed as an inset plot. For different resonant modes, the in-plane lateral mode exhibits a quality factor exceeding that of the fundamental flexural mode by four times, while the latter is conventional for liquid sensing. Unlike the fundamental flexural mode, where width variations negligibly affect quality factor, higher-order modes indicate a width-dependent quality factor that increases until reaching a limiting value. This behavior indicates a two-dimensional modal effect, which enables optimization design across both length and width scales in plate-based resonators.

The frequency domain analysis was performed to illustrate the flexural stress distribution on slender beams of the microcantilever, as shown in Fig. 2b. The magnitude of the excitation voltage was 5 V. Frequency sweeps identified the flexural, torsional, and lateral modes. Results reveal that the lateral vibration mode exhibits the highest magnitude of flexural normal stress with highly uniform longitudinal distribution along the beam. This characteristic allows enhanced self-sensing readout through piezoelectrically induced voltage measurements. Additionally, distinct frequency separation and output amplitudes between lateral and out-of-plane vibration modes mitigate mode coupling. These advantages support the adoption of the lateral-mode configuration for viscosity sensing in liquids.

### Structure optimization

**Fig. 3:**
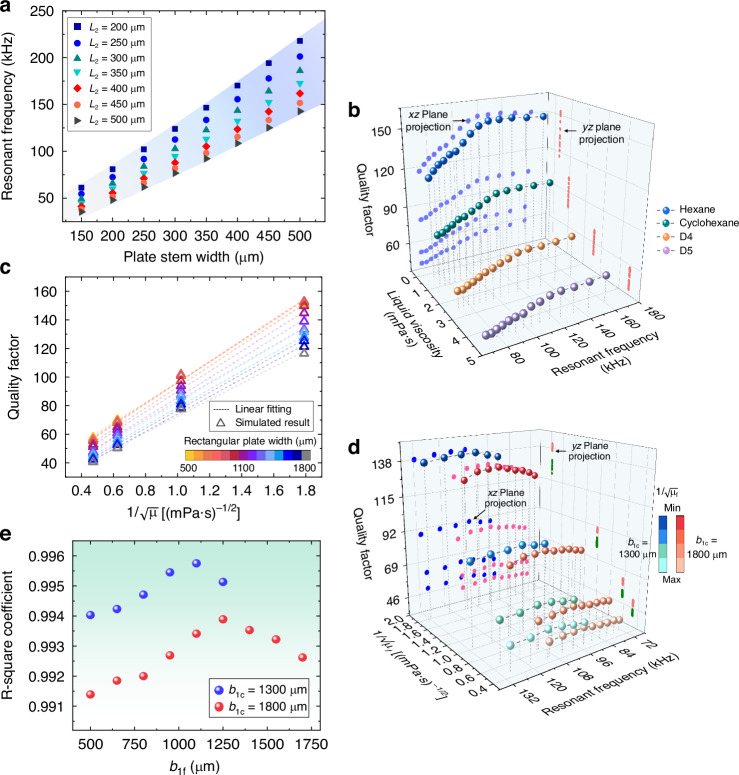
Design optimization of the microcantilever-based resonator under lateral modes. **a** Variation of the resonant frequency with the plate stem dimensions, in which the length ranges from 200 to 500 μm and the width ranges from 150 to 500 μm. **b** Quality factor as a function of resonant frequency and dynamic viscosity of liquid, for the rectangular-plate microcantilever. **c** Linear relationship between the quality factor and liquid viscosity for the rectangular-plate microcantilever. **d** Quality factor versus resonant frequency and square root of the liquid viscosity for trapezoidal-plate microcantilever with b_1c_ = 1300 μm/1800 μm and b_1f_ ranging from 500 to 1250 μm/1700 μm. **e** Dependence of the linear correlation coefficient (Quality factor-1/√*μ*_*f*_) on free side width (*b*_1f_) of the trapezoidal-plate structure for the microcantilever

The geometric analyses of the plate stem are shown in Fig. 3a, in which the width ranges from 150 to 500 μm while the length varies between 200 and 500 μm. These results indicate that the width predominantly influences the resonant frequency by affecting the modal stiffness, which shows a maximum increase of 303% at the 500 μm length. This finding also confirms the width dependence of the lateral-mode characteristics in plate-based resonators. Four liquid media with referenced viscosity and density values are listed in Table 1, which was obtained from commercially available REFPROP (Reference Fluid Properties) software. Figure 3b depicts the quality factor versus resonant frequency for the microcantilever immersed in liquid, with resonant frequency modulated by rectangular-plate widths from 500 to 1800 μm. The two-dimensional projections in the *xz*- and *yz*-planes are included. At a fixed plate width, the resonant frequency decreased by 2.97–3.15% from hexane to D5 medium, with at least one-third of the reduction observed in out-of-plane modes^27,28^. In contrast, the quality factor decreased substantially by 61.68 – 65.09%, which highlights its dominant sensitivity to liquid properties. Thus, for the resonator vibrating laterally, the inertial damping effects can be suppressed due to the minor frequency shift across liquids. The simulations investigating the influence of liquid density on resonant frequency and quality factor are provided in the Supplementary Information. These results confirm the quality factor to function as the primary indicator of viscous dissipation. By this means, a single-variable relationship between the quality factor and liquid viscosity is established, as shown in Fig. 3c. Linear fitting yields an R-square coefficient exceeding 0.99 for all tested widths of the rectangular host plate, which indicates that the proposed microcantilever displays considerable linearity with viscosity. This allows enhanced measurement accuracy through a simplified calibration model.

**Table 1:** Values of liquid density *ρ*_*ref*_ and dynamic viscosity *μ*_*ref*_ used in simulation analyses

Liquid medium	Density *ρ*_*ref*_ (g·cm^−^^3^)	Dynamic viscosity *μ*_*ref*_ (mPa·s)
Hexane	0.659	0.312
Cyclohexane	0.779	0.959
D4	0.946	2.565
D5	0.959	4.469

Further parameterization analyses for the trapezoidal host plate of the microcantilever are presented in Fig. 3d–e. The junction side width *b*_1c_ is fixed at 1300 μm with free side width *b*_1f_ ranging from 500 – 1250 μm. When the *b*_1c_ is given by 1800 μm, the free side width *b*_1f_ ranges from 500–1700 μm. The parameter sweep results are also projected onto *xz*- and *yz*-planes, separately. Reducing *b*_1f_ from maximum to minimum increases the resonant frequency by 27% for *b*_1c_ = 1300 μm, and by 33% for *b*_1c_ = 1800 μm. The quality factor shows high dependence on the junction side width, whereas its changes with the free side width can be negligible. Additionally, the linear correlation coefficient is related to the free side width of the trapezoidal plate, as shown in Fig. 3e. Actually, high-performance viscosity sensors require the resonator operating at relatively low frequencies^29^ while maintaining a high quality factor under liquid immersion. This combination enables precise electrical characterization and facilitates integration, yet represents conflicting design in conventional devices. However, in our design strategy, the resonant frequency and quality factor can be independently optimized through geometric parameters. For a specified junction side width, the free side width can be chosen to balance achieving a superior quality factor versus 1/√*μ*_*f*_ linearity, and obtaining a sufficient resonant frequency for electrical characterization. The quality factor remains nearly constant, thereby ensuring vibrational stability during lateral oscillation in viscous media. Thus, the operating frequency, quality factor and its linearity with viscosity can be simultaneously optimized for liquid sensing. Based on these simulation results, the designed dimensions of the cantilevered plate resonator are listed in Table 2.

**Table 2:** Compromised dimensional parameter of the microcantilevered plate resonator

Parameter	Value (μm)	Parameter	Value (μm)
*L*_1_	1050	*b*_2_	250
*b*_1c_	1750	*L*_3_	130
*b*_1f_	1350	*b*_3_	22
*L*_2_	320	*h*	25

## Results and discussion

### Resonator chip fabrication

**Fig. 4:**
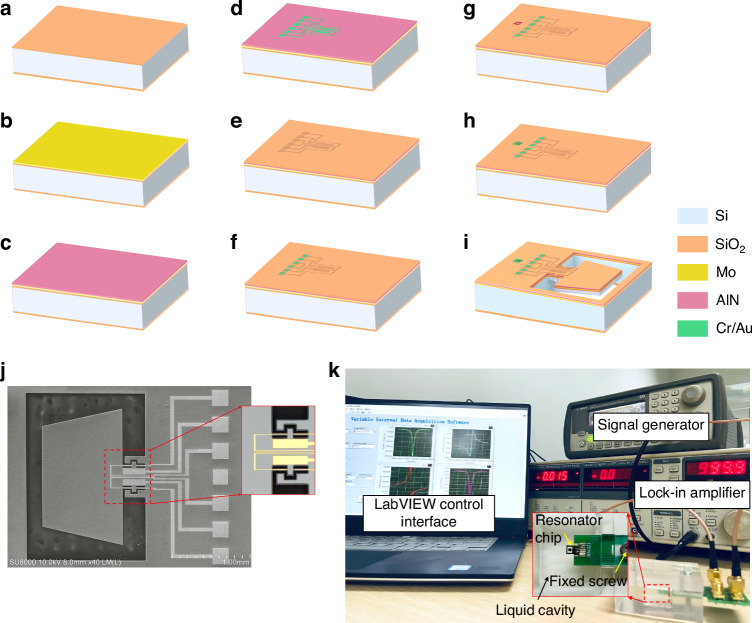
Schematic fabrication process of the resonator chip. **a** Thermal oxidization. **b** Mo film deposition. **c** AlN film deposition. **d** Metal layer sputtering. **e** LPCVD SiO_2_ layer deposition. **f** Bonding pad exposure. **g** Bottom electrode exposure. **h** Bonding pad formation of the bottom electrode. **i** Microcantilever-based resonator release. **j** Fabricated piezoelectric resonator chip, including SEM image and detailed LSCM image. **k** Experimental platform of the resonator chip for liquid sensing

The detailed fabrication process of the resonator is illustrated in Fig. 4a–i. The resonator chip was fabricated on an N-type monocrystalline silicon wafer with a substrate thickness of 500 μm, where a 350 nm SiO₂ layer was grown via thermal oxidation on both sides to serve as an insulating layer for the low-resistivity silicon substrate. Subsequently, a Mo layer with a thickness of 110 nm was deposited by magnetron sputtering as the bottom electrode. Owing to its body-centered cubic crystal structure, Mo provides crystallographic texture transfer capability to AlN thin films with hexagonal symmetry. This allows the Mo layer to function as a growth template that promotes preferentially *c*-axis oriented AlN deposition. The AlN piezoelectric film with a thickness of 1 μm was then formed by reactive magnetron sputtering, followed by deposition and patterning of Cr/Au layers (20 nm/200 nm) to define the top electrodes and electrical leads. A 240 nm low-stress SiO₂ isolation layer was deposited to cover the wafer. Afterward, the SiO₂/AlN layers were patterned to expose the electrodes, thereby metal layers were deposited and patterned to form the bonding pads for the Mo bottom electrode. The silicon substrate was then etched from the backside to a depth of 475 μm using deep reactive ion etching to create the cavity. Finally, the resonator structures were patterned and released using inductively coupled plasma etching.

The fabricated resonator chip was characterized by scanning electron microscope (SEM) imaging, as shown in Fig. 4j, with a detailed view of the plate stem and slender beam through laser scanning confocal microscopy (LSCM). The concave frames surrounding the slender beam are introduced to improve etch uniformity during the fabrication process. The overall dimensions of the resonator chip are 4.2 mm × 3.4 mm × 0.5 mm (length × width × thickness). This size is smaller than that of some reported resonators for viscosity sensing^30,31^, which improves its suitability for integration into miniaturized systems, particularly for embedded applications in portable liquid monitoring. The experimental platform is shown in Fig. 4k, where a LabVIEW interface was developed for user control. The resonator chip was bonded to the PCB board using a non-conductive epoxy resin and fixed within a customized liquid cavity. The electrical connections, including the wire bonds and bonding pads, were encapsulated with a potting adhesive for electrical isolation under the liquid immersion. The packaged device is supplied with alternating current voltage from a signal generator. A lock-in amplifier is employed to output the piezoelectric voltage signal. Through this configuration, voltage-based self-actuation and self-sensing capabilities are implemented for the resonator device. Consequently, the resonator chip can be efficiently operated for viscosity measurement within liquid media environments.

### Performance characterization

**Fig. 5:**
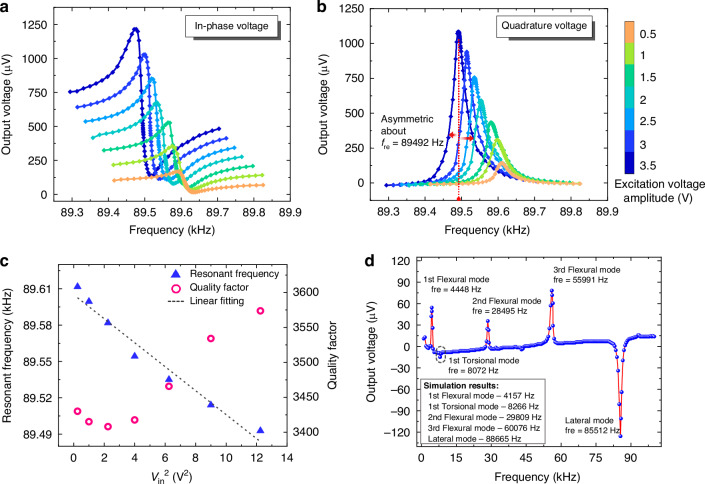
Sensing characteristics of the resonator vibrating in air and hexane. **a** Frequency response in air measured from the in-phase component of the output voltage, and its variations versus excitation voltage. **b** Frequency response in air measured from the quadrature component of the output voltage, and its variations versus excitation voltage. **c** Quantified variations in resonant frequency and quality factor versus excitation voltage amplitude in air. **d** Frequency response of the resonator vibrating in hexane

Figure 5a, b illustrates the frequency response of the resonator under varied excitation voltages in ambient air. Both resonant frequency and quality factor exhibit input-amplitude dependence. Nonlinear response of the resonator emerges when excitation exceeds 1.5 V, which can be evidenced by asymmetric quadrature voltage curves about the resonant frequency. Figure 5c quantifies resonant frequency and quality factor variations. The resonant frequency reduction results from the thermally induced decrease in the Young’s modulus^8^, where elevated excitation voltages increase the material temperature of the resonator. The quality factor reduction between 0.5 and 1.5 V can be attributed to a rise in dissipation of the vibrational energy, primarily arising from thermoelastic dissipation^32^ within the multilayered thin films of the resonator. As the excitation voltage increases in the range of 1.5–3.5 V, the enhanced electric field intensity within the AlN thin film increases the electromechanical energy conversion efficiency, which results in the improvement of the quality factor through mitigating electrical losses^33,34^. These results indicate that the piezoelectric resonator requires sufficient driving voltage to achieve preferable vibrational performance^35,36^. On the other hand, the nonlinearity behavior on the frequency response degrades the extraction accuracy of vibrational parameters during curve fitting.

Based on these results, an excitation voltage amplitude of 3 V was selected to exploit nonlinear effects while maintaining sufficient accuracy for resonant parameter extraction. To minimize the influence of temperature variations on the liquid viscosity and mechanical properties of the resonator, the experiments were conducted at a room temperature of 20 ± 0.2 °C, which is controlled by an air conditioning system. The measured frequency response in hexane across vibrational modes is presented in Fig. 5d. The resonator in lateral mode exhibits peak piezoelectrically induced voltage at its higher-order vibrational frequency. The more than 29 kHz frequency separation between this lateral mode and other out-of-plane modes ensures the purity of the mode shape for stable vibration in viscous sensing. An inset figure shows simulated resonant frequencies for flexural, torsional, and lateral modes of the fabricated resonator. Compared with the experimental results, the mean deviation of the simulated frequency is 4.91% with a maximum deviation of 7.30%. This agreement supports the validity of the fluid-structure interaction model used for resonator optimization.

**Fig. 6:**
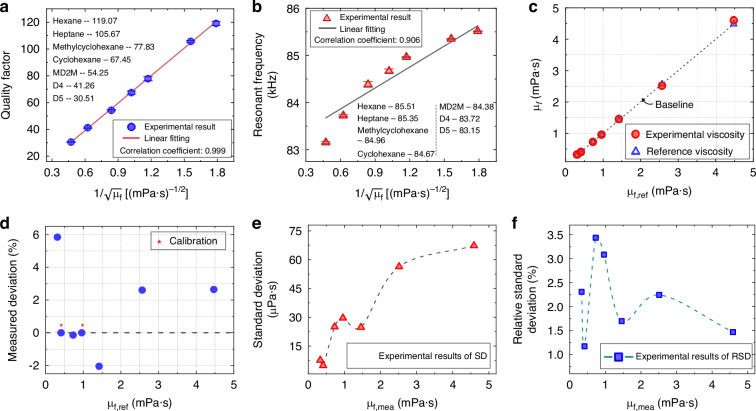
Experimental results of the resonator as a viscosity sensor. **a** Quality factor in various liquid media, with error bars indicating the standard deviation. **b** Resonant frequency in various liquid media, with error bars indicating the standard deviation. **c** Comparison of liquid viscosities based on experimental results and reference values. **d** Measurement deviation of the resonator for the liquid viscosity. **e** Standard deviation (SD) results of viscosity measurements in liquid media. **f** Relative standard deviation (RSD) results of viscosity measurements in liquid media

In addition to the liquids listed in Table 1, heptane (density of 0.684 g·cm^−^^3^, viscosity of 0.411 mPa·s), methylcyclohexane (density of 0.769 g·cm^−^^3^, viscosity of 0.729 mPa·s) and MD2M (density of 0.854 g·cm^−^^3^, viscosity of 1.420 mPa·s) were added to the experiments. The quality factor and resonant frequency were tested at least ten times in each liquid to calculate the average value for viscosity determination. As shown in Fig. 6a–b, an excellent linear correlation exists between the quality factor and the square root of viscosity, with R-square coefficient reaching 0.999. The resonant frequency exhibits comparatively weaker linearity with the viscosity term. These relationships enable direct quantification of viscosity variations through resonance parameters and significantly simplify the calibration procedure. In contrast, conventional methods typically exhibit nonlinear response dependencies on liquid viscosity^30,37^, which are accompanied with a limitation that necessitates density calibration for viscosity measurement. Owing to the viscosity-dependence linearity, the measurement sensitivity is defined as the linearity slope with a value of 68 (mPa·s)^1/2^.

Building upon the observation that both the quality factor and resonant frequency vary inversely with the square root of viscosity, and the resonance parameters can be characterized by Eq. (1). By this means, the derivation of an explicit viscosity measurement function can be presented as Eq. (2). This advancement enables the direct determination of liquid viscosity while eliminating the conventional density calibration requirement^31,38^, thereby overcoming a fundamental limitation to existing methods in high-precision viscosity measurement of liquids.

1 $${Q}_{L}\propto {{\mu }_{L,mea}}^{-\frac{1}{2}}{(2\pi {f}_{R})}^{-\frac{3}{2}}$$

2 $${\mu }_{L,mea}={({{Q}_{L}}^{-1}+{K}_{1})}^{2}{{f}_{R}}^{-3}{K}_{2}$$

where *μ*_*L,mea*_ is the measured value of the liquid viscosity, *K*_1_ and *K*_2_ are coefficients determined during the calibration process, *Q*_*L*_ and *f*_*R*_ are the measured quality factor and resonant frequency under liquid immersion, respectively.

The fabricated resonator served as a viscosity sensor with calibrated coefficients *K*_1_ = 1.08 × 10^−^^3^ and *K*_2_ = 2.30 × 10^18^ mPa·s·Hz^3^. As shown in Fig. 6c, d, the measured viscosities across various liquids indicated outstanding agreement with reference viscosity values, yielding a mean absolute relative deviation of 2.65% and a maximum deviation of 5.84%. Beyond accuracy, the viscosity measurement stability of the resonator is critical for ensuring consistent results in liquid monitoring applications, yet it is seldom quantified in the existing studies. In this work, the viscosity measurement stability was evaluated by standard deviation (SD) and relative standard deviation (RSD, defined as SD/mean viscosity). Figure 6e, f reveals that the proposed resonator achieves 67 μPa·s for SD and 3.43% for RSD under worst-case conditions. This performance approaches the ±3% RSD specification of the commercial MiniVisc 3000 viscometer (Spectro Scientific Inc.). In addition, the resolution was estimated from the standard deviation in the resonant frequency and quality factor by Eq. (3):

3 $$\Delta {\mu }_{L}=\sqrt{{\left(\frac{\partial {\mu }_{L}}{\partial {Q}_{L}}\Delta {Q}_{L}\right)}^{2}+{\left(\frac{\partial {\mu }_{L}}{\partial {f}_{R}}\Delta {f}_{R}\right)}^{2}}=\sqrt{{\left(-\frac{2\zeta {K}_{2}}{{Q}_{L}^{2}{f}_{R}^{3}}\Delta {Q}_{L}\right)}^{2}+{\left(-\frac{3{\zeta }^{2}{K}_{2}}{{f}_{R}^{4}}\Delta {f}_{R}\right)}^{2}}$$

where Δ*μ*_*L*_ is the estimated resolutions for viscosity, *ζ* satisfies the function of *ζ* = 1/*Q*_*L*_ + *K*_1_, Δ*Q*_*L*_ and Δ*f*_*R*_ are fluctuations determined by the standard deviations of the quality factor and resonant frequency of the resonator, respectively. The value obtained for the model solutions is below 69 μPa·s.

Table 3 summarizes the main performances of the proposed laterally vibrating resonator for liquid viscosity sensing, alongside comparative data from published literature. Through the high-linearity design strategy, the experimental results validate superior comprehensive performances of the resonator in direct viscosity determination, with over 34% reduction in measurement error compared to state-of-the-art approaches. To further extend the measurement range for highly viscous liquids, advanced piezoelectric materials such as potassium sodium niobate (KNN) films^39^, and scandium-substituted AlN (ScAlN) films^40^ show considerable promise. The temperature variation is an important factor that influences the sensing performance of the resonator. The dynamic viscosity of most Newtonian liquids decreases exponentially with increasing temperature^41^. Simultaneously, the resonant frequency of silicon-based resonators typically exhibits a negative shift with temperature, primarily due to the temperature dependence of the Young’s modulus of silicon^8^. These concerns were largely mitigated by conducting the experiments in a temperature-controlled environment. Additionally, a temperature compensation algorithm could be employed to further enhance the measurement accuracy of the resonator in future work. This strategy would mainly correct for the temperature-induced variation in the Young’s modulus of the resonator under ambient conditions.

**Table 3:** Performance comparison with previous reported resonators for liquid viscosity sensing

	Ref.^21^	Ref.^31^	Ref.^44^	Ref.^45^	This work
Accuracy	10.1%	5.78%	5.35%	4%	2.65%
Range	1–10.5 mPa·s	0.2–1.0 mPa·s	1.8–2.0 mPa·s	0.9–6.6 mPa·s	0.3–4.5 mPa·s
Stability	3.2 mPa·s	–	–	–	67 μPa·s/ 3.43%
Sensitivity	–	–	7.31 (mPa·s)^−^^1^	–	68 (mPa·s)^1/2^ [~18.53 (mPa·s)^−^^1^]
Actuation/ sensing principle	Photothermal/ optical	Magnetic/ piezoresistive	Piezoelectric	Photothermal/ optical	Piezoelectric

By integrating lateral-mode vibration with voltage-based electrical characterization, the fabricated resonator achieves precise, independently measurable fluidic parameters while maintaining operational simplicity. These investigations establish a potential foundation for developing high-performance piezoelectric resonators, particularly for liquid monitoring applications in industrial diagnostics and environmental monitoring. In addition, the design method featuring a lateral vibration mode with high-linearity response and self-sensing capability can be extended to other piezoelectric resonant sensors. This includes devices like pressure sensors^42^ and accelerometers^43^ for achieving highly accurate and integrated measurements.

## Conclusion

This work presents a novel piezoelectric laterally vibrating resonator for direct measurement of liquid viscosity. The resonator was designed as a cantilevered dual-plate structure featuring wide steps, which achieved functional separation of fluid interaction domains while allowing self-actuated and self-sensing capabilities. Fluid-structure interaction modeling was established to guide geometric optimization to enhance the quality factor and its viscosity-dependence linearity. Experimental characterization of the fabricated AlN-resonator confirmed a highly linear model between its resonance parameters and the viscosity of various test liquids. This condition enabled direct viscosity determination without the typical requirement for coupled density calibration, which reached a high accuracy of 2.65%. In addition, the viscosity measurement stability showed a maximum standard deviation of 67 μPa·s. Our results demonstrated that the high-performance resonator was achieved by balancing density-independent viscosity determination with fully electrical characterization at a moderate resonant frequency. These advances contribute to addressing industrial needs for high-precision viscosity sensing in liquid monitoring applications.

## Supplementary information

Supplementary Information
