# Supplementary material for: Piezoelectric stepped-plate resonators vibrating at lateral modes for direct viscosity determination in liquids
Source: Microsyst Nanoeng. 2026 Apr 7;12:122. doi: 10.1038/s41378-025-01135-7 (PMC13057218; doi:10.1038/s41378-025-01135-7)
Supplement: Supplementary file 1 — Supplementary Information [file 41378_2025_1135_MOESM1_ESM.docx]

**Piezoelectric stepped-plate resonators vibrating at lateral modes for direct viscosity determination in liquids**

**[SUPPLEMENTARY MATERIAL]**

Linya Huang^1,2^, Dejiang Lu^1,3,4,^*, Xiangguang Han^1,3,4^, Heping Wu^2^, Wei Li^1,3^, Gang Niu^1,2,*^, Kaifei Wang^5,*^, Ping Yang^1^ , Wei Ren^1,2^, Libo Zhao^1,3,4^

^1^State Key Laboratory for Manufacturing Systems Engineering, International Joint Laboratory for Micro/Nano Manufacturing and Measurement Technologies, Xi’an Jiaotong University, 710049 Xi’an, China

^2^Electronic Materials Research Laboratory, Key Laboratory of the Ministry of Education, School of Electronic Science and Engineering, Xi’an Jiaotong University, 710049 Xi’an, China

^3^School of Instrument Science and Technology, Xi’an Jiaotong University, 710049 Xi’an, China

^4^State Industry-Education Integration Center for Medical Innovations, International Joint Laboratory for Micro/Nano Manufacturing and Measurement Technologies, Shaanxi Innovation Center for Special Sensing and Testing Technology in Extreme Environments, Shaanxi Provincial University Engineering Research Center for Micro/Nano Acoustic Devices and Intelligent Systems, Xi’an Jiaotong University, Xi’an 710049, China.

^5^Department of Emergency, The First Affiliated Hospital of Xi’an Jiaotong University, Xi’an 710049, China.

*Corresponding Authors: Dejiang Lu (djlu@mail.xjtu.edu.cn), Gang Niu (gangniu@mail.xjtu.edu.cn), or Kaifei Wang (kaifeiw@xjtufh.edu.cn)

**Materials and methods**

**Structure optimization**

The influence of liquid density on the resonant frequency and quality factor was investigated using cyclohexane as the medium. Simulations were conducted for the resonator with seven different widths of the rectangular host plate. To isolate the density-dependent characteristics of the lateral-mode resonator, the viscosity of the cyclohexane was held constant at 0.959 mPa·s while the density was varied from 0.579 to 0.979 g/cm^3^. Fig. S1 shows a highly linear relationship between the resonant frequency and liquid density for all rectangular-plate widths, with R-square coefficients exceeding 0.999. The resonant frequency decreases by 3.86 – 4.05% across the liquid density range, with an absolute shift of 3.01 – 5.83 kHz. This reduction is attributed to the increased fluid mass loading on the resonator as the liquid density increases. In contrast, the quality factor exhibits minor fluctuations with changing liquid density, with the absolute change ranging from 0.21 to 2.40. Notably, for plate widths exceeding 1200 μm, the fluctuation in quality factor remains low, which indicates that increasing the host plate width enhances the independence of the quality factor from liquid density. These results demonstrate that the resonant frequency is primarily sensitive to liquid density, while the quality factor remains largely independent of the liquid density. This condition verifies the use of a single-variable relationship between the quality factor and liquid viscosity for sensing purposes.

**
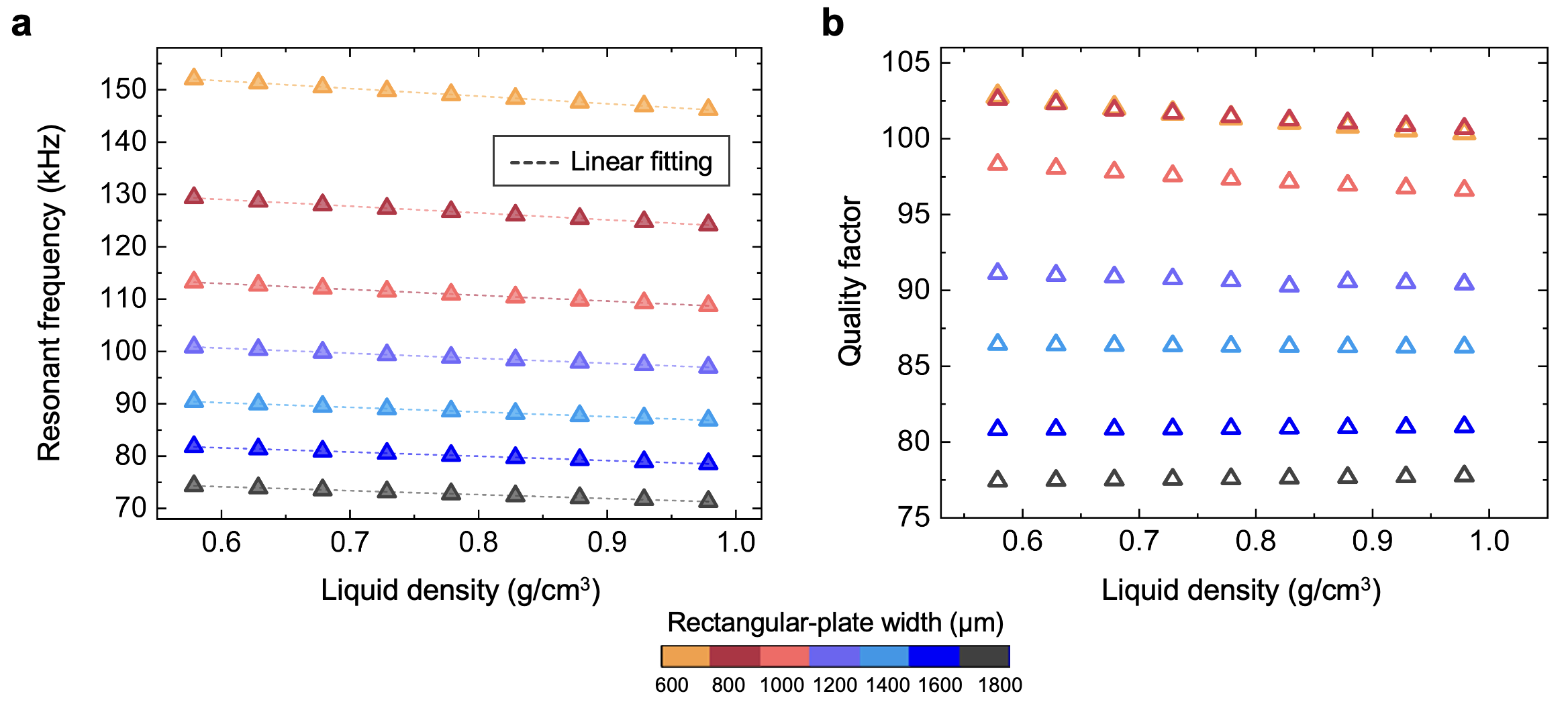
**

**Fig. S1. Simulation results of the microcantilever-based resonator with different rectangular-plate widths and under varying liquid densities. a** Resonant frequency of the resonator versus liquid density. **b** Quality factor of the resonator versus liquid density.
